# The Prevalence of Y Chromosome Microdeletions in Iranian Infertile Men with Azoospermia and Severe Oligospermia 

**DOI:** 10.22074/cellj.2016.4863

**Published:** 2016-12-21

**Authors:** Fahimeh Asadi, Mohammad Ali Sadighi Gilani, Azadeh Ghaheri, Javad Roodgar Saffari, Mohammadreza Zamanian

**Affiliations:** 1 Department of Molecular and Cellular Sciences, Faculty of Advanced Sciences and Technology, Pharmaceutical Sciences Branch, Islamic Azad University, Tehran, Iran; 2 Department of Andrology, Reproductive Biomedicine Research Center, Royan Institute for Reproductive Biomedicine, ACECR, Tehran, Iran; 3 Department of Epidemiology and Reproductive Health, Reproductive Epidemiology Research Center, Royan Institute for Reproductive Biomedicine, ACECR, Tehran, Iran; 4 Department of Genetics, Reproductive Biomedicine Research Center, Royan Institute for Reproductive Biomedicine, ACECR, Tehran, Iran

**Keywords:** Male Infertility, Y Chromosome, Oligospermia, Azoospermia

## Abstract

**Objective:**

Microdeletions of the Y chromosome long arm are the most common molecular genetic causes of severe infertility in men. They affect three regions including azoospermia factors (AZFa, AZFb and AZFc), which contain various genes involved in spermatogenesis. The aim of the present study was to reveal the patterns of Y chromosome microdeletions in Iranian infertile men referred to Royan Institute with azoospermia/ severe oligospermia.

**Materials and Methods:**

Through a cross-sectional study, 1885 infertile men referred to Royan Institute with azoospermia/severe oligospermia were examined for Y chromosome microdeletions from March 2012 to March 2014. We determined microdeletions of the Y chromosome in the AZFa, AZFb and AZFc regions using multiplex Polymerase chain reaction and six different Sequence-Tagged Site (STS) markers.

**Results:**

Among the 1885 infertile men, we determined 99 cases of Y chromosome microdeletions (5.2%). Among 99 cases, AZFc microdeletions were found in 70 cases (70.7%); AZFb microdeletions in 5 cases (5%); and AZFa microdeletions in only 3 cases (3%). AZFbc microdeletions were detected in 18 cases (18.1%) and AZFabc microdeletions in 3 cases (3%).

**Conclusion:**

Based on these data, our results are in agreement with similar studies from other regions of the world as well as two other recent studies from Iran which have mostly reported a frequency of less than 10% for Y chromosome microdeletions.

## Introduction

Infertility is defined as a failure of fertilization in a couple after a year of regular unprotected sexual intercourse (1). Infertility is a major health and reproductive problem that affects 10 to 15% of couples, of which nearly 50% of the cases are due to factors affecting the male (2). 

Different etiologies have been identified for male infertility such as varicocele, erectile dysfunction, ejaculation failure, obstruction of the spermatic duct, hormonal imbalance and genetic factors, such as deletions or mutations in the genes responsible for spermatogenesis (3). In 50% of cases male infertility is idiopathic, whereas genetic factors play a role in about 10% of cases (4). 

The male sex-determining region (SRY) is located on the short arm of the Y chromosome (Yp11) (5), while important genes involved in spermatogenesis are located on the proximal part of its long arm (Yq11). This area of chromosome Y is recognized as the azoospermia factor (AZF) region and is divided into the AZFa, AZFb and AZFc sub regions. The most important genes in the AZF region are *USP9Y, DBY, UTY, TB4Y* in the AZFa, *EIF1AY, PRY, TTY2, RBMY* in the AZFb and *DAZ1, DAZ2, BPY2, PRY* and *CDY* in the AZFc sub regions (6). 

The most common microdeletion is seen in the AZFc sub region and is accompanied by DAZ gene deletion and moderate to severe oligozoospermia (7,8), while microdeletions in the AZFa and AZFb sub regions have been correlated with azoospermia. In general, deletions in the AZF region have been associated with altered sperm parameters and testicular histological characteristics which range from Sertoli cell only syndrome (SCOS) to hypospermatogenesis (2). Microdeletions in the AZF region are the most common defects in spermatogenesis. They can be detected through molecular methods (9) and are reported in 5-10% of infertile men (10). In most studies, deletions of AZFc region are the most frequent, followed by deletions in the AZFb and AZFa regions (11). 

Application of assisted reproductive technologies (ART), such as *in vitro* fertilization (IVF) and intracytoplasmic sperm injection (ICSI), involving men with Yq microdeletions will increase the risk of infertility and maybe other diseases in their male offspring (12,14). Totonchi et al. (15) showed that the incidence of these microdeletions among Iranian infertile men was 5.1% and Zaimy et al. (16) also indicated that the incidence of Y chromosome microdeletions in Iranian infertile men was 5%. 

The aim of present study was to evaluate Y chromosome microdeletions in Iranian infertile male population based on STS markers provided latest EAA/EMQN guideline (2013) which was the first large scale study in this field. 

## Materials and Methods

### Patients

This cross-sectional study was approved by the Ethics Committee of the Royan Institute, and written informed consent was obtained from all participants. One thousand and eight hundred eighty five infertile men referred to the Royan Institute between March 2012 and March 2014 were included. Complete semen analysis according to normal standard parameters using World Health Organization (WHO) criteria was performed in the andrology laboratory of the Royan Institute to check semen volume, pH, sperm concentration, motility and morphology. On the basis of their seminal profile, infertile cases were classified as azoospermia (zero sperm count) or severe oligozoospermia (sperm count less than 5 million per ml). Hormonal analyses, including follicle stimulating hormone (FSH), luteinizing hormone (LH) and testosterone, were performed and anatomical integrity of the male genital system was evaluated through urological examination. 

Infertile men with congenital absence of vas deferens, obstructive azoospermia, gonadal abnormalities, varicocele or a history of cryptorchidism, chemotherapy, radiation therapy, orchitis, testicular injury or abnormal karyotypes were excluded from the study. 

### Detection of Y chromosome microdeletions

Genomic DNA was extracted from peripheral blood lymphocytes using a PAX gene kit (Qiagen, Germany) following the manufacturer’s protocol. Extracted DNA was stored at -20˚C. Six STS markers on the AZF region of Yq11 were used for the detection of microdeletions according to the European Academy of Andrology, the European Molecular Genetics Quality Network (EAA, EMQN) (17). The STS markers and related information are summarized in Table 1. SRY and *ZFX/ZFY* were used as internal controls for Y and X/Y chromosome detection respectively.

Multiplex polymerase chain reaction (PCR) reactions were prepared in two different mixes; A and B. Each of the mixes contained 50 mM KCL, 10 mM Tris-HCL pH=8, 200 µM dNTP, 2 mM MgCl_2_ and 1 µL Taq DNA polymerase (CinnaGen, Iran) in 25 µL final volume. For the positive controls we used male and female blood samples, as well as no-template samples (blank) as the negative control. 

Amplification conditions were 4 minutes initial denaturation at 95˚C followed by 35 cycles of 30 seconds denaturation at 94˚C, 90 seconds annealing at 57˚C and 60 seconds elongation at 72˚C, followed by a final elongation step of 7 minutes at 72˚C. Finally PCR products were analyzed on 3% agarose gel containing SYBR Green (ABM, Canada). Data analysis was done using SPSS version 17.

## Results

### Patients

In this study 1885 infertile men with azoospermia/ severe oligospermia were evaluated for possible Y chromosome microdeletions. Among them, 99 cases (5.2%) were diagnosed with microdeletions in different AZF regions. The mean age of the cases was 34.1 ± 6.4 years (range: 22-45 years) at study inclusion. 

### Y chromosome microdeletion analysis

**Fig.1:**
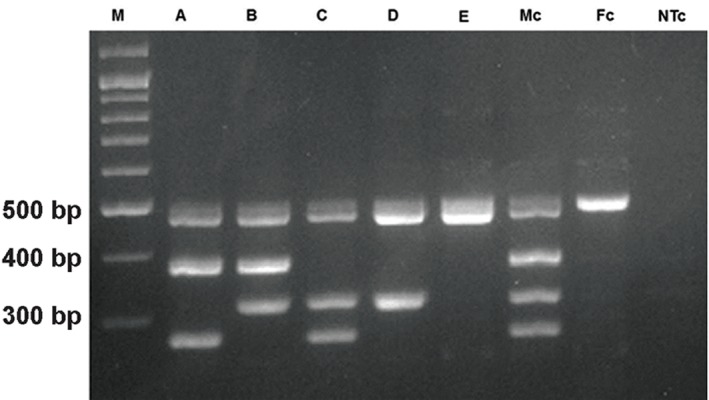
Agarose gel electrophoresis results of Mix A. M; 100 bp Ladder, A-E. Samples with deletions in AZFa, AZFb, AZFc, AZFbc, and AZFabc respectively, Mc; Male control, Fc; Female control, and NTc; No template control.

**Fig.2:**
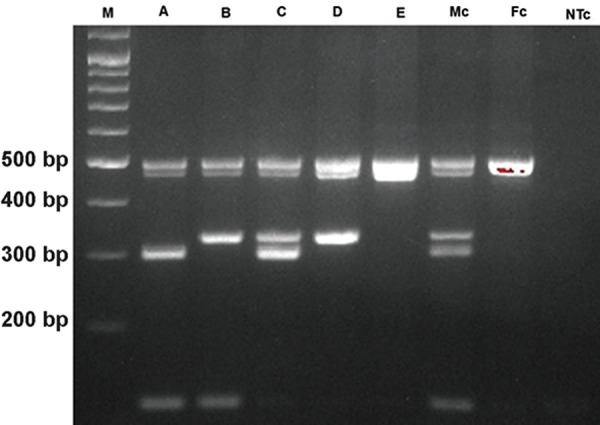
Agarose gel electrophoresis results of Mix B. M; 100 bp Ladder, A-E. Samples with deletions in AZFa, AZFb, AZFc, AZFbc and AZFabc respectively, Mc; Male control, Fc; Female control, NTc; No template control.

Most Yq microdeletions (70 out of 99) were found in the AZFc region (70.7%), while AZFa and AZFb microdeletions were found in 3 (3%) and 5 cases (5%), respectively (Fig.1,2). Extended microdeletions including AZFbc and AZFabc were also detected in 18 (18.1%) and 3 (3%) of the cases respectively. 

**Table 1:** STS markers, primer sequence and product size for Y chromosome microdeletions


	STS	AZF region	Product size (bp)	Primer sequence (5ˊ-3ˊ)

Mix A	sY86	AZFa	320	F: GTG ACA CAC AGA CTA TGC TTC
				R: ACA CAC AGA GGG ACA ACC CT
	sY127	AZFb	274	F: GGC TCA CAA ACG AAA AGA AA
				R: CTG CAG GCA GTA ATA AGG GA
	sY254	AZFc	400	F: GGG TGT TAC CAG AAG GCA AA
				R: GAA CCG TAT CTA CCA AAG CAG C
	ZFX/ZFY	-	-	F: ACC RCT GTA CTG ACT GTG ATT ACA C
				R: GCA CYT CTT TGG TAT CYG AGA AAG T
	sY14	SRY	-	F: GAA TAT TCC CGC TCT CCG GA
				R: GCT GGT GCT CCA TTC TTG AG
Mix B	sY84	AZFa	326	F: AGA AGG GTC CTG AAA GCA GGT
				R: GCC TAC TAC CTG GAG GCT TC
	sY134	AZFb	301	F: GTC TGC CTC ACC ATA AAA CG
				R: ACC ACT GCC AAA ACT TTC AA
	sY255	AZFc	126	F: GTT ACA GGA TTC GGC GTG AT
				R: CTC GTC ATG TGC AGC CAC
	ZFX/ZFY	-	-	F: ACC RCT GTA CTG ACT GTG ATT ACA C
				R: GCA CYT CTT TGG TAT CYG AGA AAG T
	sY14	SRY	-	F: GAA TAT TCC CGC TCT CCG GA
				R: GCT GGT GCT CCA TTC TTG AG


STS; Sequence-Tagged Site and AZF; Azoospermia factors.

### Semen analysis

**Fig.3:**
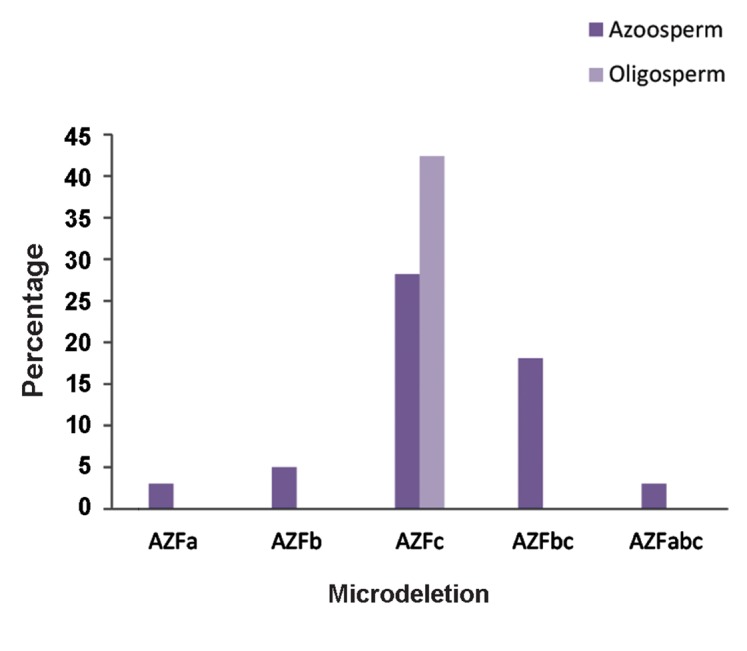
Percentage of patients with a Y chromosome microdeletions in terms of their sperm.

Among the 99 cases with microdeletions, 71 men (71.7%) were suffering from azoospermia while 28 cases (28.2%) had severe oligospermia. In the azoospermic group there were 42 cases (59.1%) with microdeletion in the AZFc region, 18 cases (25.3%) with AZFbc microdeletion, 5 cases (7%) with AZFb microdeletion, 3 cases (4.2%) with combined deletion of AZFabc, and 3 cases (4.2%) with AZFa microdeletion. All men in the severe oligospermic group (28 cases; 100%) had AZFc microdeltions (Table 2,Fig.3). 

All men with microdeletions in AZFa, AZFb, AZFbc and AZFabc were cases of non-obstructive azoospermia. Our results show that only cases with microdeletion in the AZFc region had evidence of sperm production. 42 cases (60%) of the 70 cases with microdeletions in the AZFc region had azoospermia and 28 (40%) had severed oligospermia. 

**Table 2:** The number and percentage of cases with Y chromosome micro-deletions in terms of their sperm count


Microdeletion region	Patients		Spermogram	
	n	%	Azoo.	Oligo.

AZFa	3	3	3	0
AZFb	5	5	5	0
AZFc	70.7	70	42	28
AZFbc	18.1	18	18	0
AZFabc	3	3	3	0
Total	5.2	99	71	28


### Hormone analysis

Among the 99 infertile men with AZF microdeletions referred to take part in the Royan Institute hormonal analysis, normal LH, FSH and testosterone levels were observed in 36 men (36.3%) while 22 men (22.2%) showed high FSH levels, 2 men (2%) had high LH levels, and 14 men (14.1%) had high levels of both LH and FSH. 

In 9 men (9%) decreased levels of testosterone were observed, of whom 2 had a high level of FSH, and 2 had high levels of both LH and FSH. 

## Karyotype analysis

Karyotype analysis was performed on the 99 cases with Y chromosome microdeletions. A normal male karyotype (46XY) was observed in 89 of the cases (89.8%) while in 10 cases small changes were observed in their karyotypes (Table 3). 

**Table 3:** Chromosomal changes in patients with AZF microdeletions


AZF microdeletion type	Karyotype

AZFbc	45,X,[7]/46,X,inv(Y)(q11.2q12)[8]
AZFc, Par.a	46,X,der(Y)del(Y)t(Y22)(p10;p10)
AZFbc, Par.a	46X del(Y)
AZFbc	46,X,del(Y)(q11.2)
AZFabc	46,X,del(Y)(q11.222)
AZFbc	46,X.idic(Y)(q11.2)[9]/45,X[9]
AZFbc	46,X,idic(Y)(q11.22)[13]/45,X[2]
AZFbc	46XX,idic(Y)(q11.22)
AZFbc	46,X,idic(Y)(q11.22)[12]/45,X[3]
AZFbc	46,X,idic(Y)(q11.22)[9]/45,X[5]/46X^+^mar[2]


A few patients showed some degrees of chromosomal changes mostly as mosaicism.

## Discussion

Deletions in the AZF region occur in the euchromatin part of the Yq chromosome and can lead to damage in the genes responsible for spermatogenesis such as *DBY* and *DFFRY* in the AZFa sub-region, *RBMY, PRY* and *CDY2* in the AZFb sub-region and finally *BPY2, CDY, DAZ, CSPG4LY* and *GOLGAZLY* in the AZFc sub- region. These microdeletions are created by intra- chromosomal recombination events between large homologous repetitive sequences (18) and are the second most common genetic causes for spermatogenic failure and male infertility after Klinefelter’s syndrome (16). Tiepolo and Zuffardi (19) were the first to discover the association between Yq deletions and spermatogenic failure, but since then many studies have confirmed this correlation (4, 20-26). Previous studies have described an incidence of 1-55% for Yq microdeletions in infertile men while most have reported a prevalence of less than 10% (27). Microdeletions in AZFc region of the Y chromosome are the most common prevalent molecular genetic problem that can lead to male infertility (28).

In Iran, several studies have determined the frequency of AZF microdeletions among infertile men. Omrani et al. (29) reported a 24.2% prevalence for Yq microdeletions among 99 infertile azoospemic men from Northwest of Iran, while Malekasgar and Mombaini (30) reported a frequency of about 50% for microdeletions among 50 azoospermic/oligospermic infertile men in South of Iran, a level much higher than other report. The higher frequency reported by them can be related to their lower sample size. In another study, Mirfakhraie et al. (31) found a frequency of Yq microdeletions of 12% among 100 Iranian azoospermic infertile men with AZFb microdeletion as the most prevalent form. Totonchi et al. (15) observed a frequency of 5.06% for the prevalence of Yq microdeletions among 185 Iranian infertile men, a finding which was confirmed by Zaimy et al. (16) who also observed prevalence of 5%. During the present study, we evaluated 1885 infertile men referred to the Royan Institute and found a frequency of 5.4% for Yq microdeltions, a finding which is completely compatible with those of Totonchi et al. (15) and Zaimy et al. (16). It should be noted that the newest EAA/EMQN best practice guidelines for the molecular diagnosis of Y-chromosomal microdeletions, published in 2013, reported a 24.2% prevalence for microdeletion among Iranian infertile men. This finding seems to be very different from most existing evidence in the current literature, including our own results (11). Part of the variation in the reported frequencies of Yq microdeletion may be due to differences in ethnicity, composition of the sample size and study population and finally the implication of STS markers. 

The EAA/EMQN best practice guideline suggests the use of 6 STS markers (2 STS for each region) which enables the detection of all clinically relevant deletions (11). It seems that use of various markers could cause part of the difference between the reports for prevalence of Y chromosome microdeletion among Iranian infertile men. For example, Omrani et al. (29) used 20 STS markers and reported an prevalence of 24.4% for microdeletions which is quite close to the EAA/EMQN report. However, the present study, which used the same EAA/EMQN STS markers observed a much lower frequency for AZF microdeltion compared to EAA/EMQN report, 5.4 versus 24.2%. Finally, among the sub regions, we detected AZFc microdeletion as the most prevalent 70.7%) which is also compatible to previous reports (2,4,32). 

## Conclusion

Among 1885 azoospermic/severe oligospermic men referred to Royan institute, we detected 99 (5.2%) cases of chromosome Y microdeletions including 70 (70.7%) of AZFc, 5 (5%) of AZFb and 3 (3%) of AZFa. These data are in agreement with previous results from the worldwide studies which have mostly reported a frequency of less than 10% for AZF microdeletions. Therefore we may postulate a similar pattern for frequency of AZF microdeletions among Iranian infertile men to the other world regions with a total frequency of 5.2% and AZFc as the most common one. 

## References

[1] Friel A, Houghton JA, Maher M, Smith T, Noël S, Nolan A (2001). Molecular detection of Y chromosome microdeletions: an Irish study. Int J Androl.

[2] Foresta C, Moro E, Ferlin A (2001). Y chromosome microdeletions and alterations of spermatogenesis. Endocr Rev.

[3] Mitra A, Dada R, Kumar R, Gupta NP, Kucheria K, Gupta SK (2008). Screening for Y-chromosome microdeletions in infertile Indian males: utility of simplified multiplex PCR. Indian J Med Res.

[4] Reijo R, Lee TY, Salo P, Alagappan R, Brown LG, Rosenberg M (1995). Diverse spermatogenic defects in humans caused by Y chromosome deletions encompassing a novel RNA-binding protein gene. Nat Genet.

[5] Raicu F, Popa L, Apostol P, Cimpomeriu D, Dan L, Ilinca E (2003). Scrreening for microdeletions in human Y chromosome--AZF candidate genes and male infertility. J Cell Mol Med.

[6] Foresta C, Ferlin A, Moro E (2000). Deletion and expression analysis of AZFa genes on the human Y chromosome revealed a major role for DBY in male infertility. Hum Mol Genet.

[7] Lahn BT, Page DC (1997). Functional coherence of the human Y chromosome. Science.

[8] Thangaraj K, Gupta NJ, Pavani K, Reddy AG, Subramainan S, Rani DS (2003). Y chromosome deletions in azoospermic men in India. J Androl.

[9] Simoni M, Bakker E, Eurlings MC, Matthijs G, Moro E, Müller CR (1999). Laboratory guidelines for molecular diagnosis of Y-chromosomal microdeletions. Int J Androl.

[10] Sadeghi-Nejad H, Farrokhi F (2007). Genetics of azoospermia: current knowledge, clinical implications, and future directions.Part II: Y chromosome microdeletions. Urol J.

[11] Krausz C, Hoefsloot L, Simoni M, Tüttelmann F (2014). European Academy of Andrology; European Molecular Genetics Quality Network.EAA/EMQN best practice guidelines for molecular diagnosis of Y-chromosomal microdeletions: state-of-the-art 2013. Andrology.

[12] Page DC, Silber S, Brown LG (1999). Men with infertility caused by AZFc deletion can produce sons by intracytoplasmic sperm injection, but are likely to transmit the deletion and infertility. Hum Repord.

[13] Kühnert B, Gromoll J, Kostova E, Tschanter P, Luetjens CM, Simoni M (2004). Case report: natural transmission of an AZFc Y-chromosomal microdeletion from father to his sons. Hum Repord.

[14] Kamischke A, Gromoll J, Simoni M, Behre HM, Nieschlag E (1999). Transmission of a Y chromosomal deletion involving the deleted in azoospermia (DAZ) and chromodomain (CDY1) genes from father to son through intracytoplasmic sperm injection: case report. Hum Repord.

[15] Totonchi M, Mohseni Meybodi A, Borjian Boroujeni P, Sedighi Gilani M, Almadani N, Gourabi H (2012). Clinical data for 185 infertile Iranian men with Y-chromosome microdeletion. J Assist Reprod Genet.

[16] Zaimy MA, Kalantar SM, Sheikhha MH, Jahaninejad T, Pashaiefar H, Ghasemzadeh J (2013). The frequency of Yq microdeletion in azoospermic and oligospermic Iranian infertile men. Iran J Reprod Med.

[17] Simoni M, Bakker E, Krausz C (2004). EAA/EMQN best practice guidelines for molecular diagnosis of y-chromosomal microdeletions.State of the art 2004. Int J Androl.

[18] Repping S, Skaletsky H, Lange J, Silber S, Van Der Veen F, Oates RD (2002). Recombination between palindromes P5 and P1 on the human Y chromosome causes massive deletions and spermatogenic failure. Am J Hum Genet.

[19] Tiepolo L, Zuffardi O (1976). Localization of factors controlling spermatogenesis in the nonfluorescent portion of the human Y chromosome long arm. Hum Genet.

[20] Vogt PH (2004). Molecular genetics of human male infertility: from genes to new therapeutic perspectives. Curr Pharm Des.

[21] Shinka T, Nakahori Y (1996). The azoospermic factor on the Y chromosome. Acta Paediatr Jpn.

[22] Silber SJ, Alagappan R, Brown LG, Page DC (1998). Y chromosome deletions in azoospermic and severely oligozoospermic men undergoing intracytoplasmic sperm injection after testicular sperm extraction. Hum Repord.

[23] Chandley AC, Cooke HJ (1994). Human male fertility--Y-linked genes and spermatogenesis. Hum Mol Genet.

[24] Navarro-Costa P, Gonçalves J, Plancha CE (2010). The AZFc region of the Y chromosome: at the crossroads between genetic diversity and male infertility. Hum Reprod Update.

[25] Walsh TJ, Pera RR, Turek PJ (2009). The genetics of male infertility. Semin Reprod Med.

[26] Li Z, Haines CJ, Han Y (2008). "Micro-deletions" of the human Y chromosome and their relationship with male infertility. J Genet Genomics.

[27] Poongothai J, Gopenath TS, Manonayaki S (2009). Genetics of human male infertility. Singapore Med J.

[28] Yamada K, Fujita K, Quan J, Sekine M, Kashima K, Yahata T (2010). Increased apoptosis of germ cells in patients with AZFc deletions. J Assist Reprod Genet.

[29] Omrani MD, Samadzadae S, Bagheri M, Attar K (2006). Y chromosome microdeletions in idiopathic infertile men from west Azarbaijan. Urol J.

[30] Malekasgar AM, Mombaini H (2008). Screening of 'Y'chromosome microdeletions in Iranian infertile males. J Hum Repord Sci.

[31] Mirfakhraie R, Mirzajani F, Kalantar SM, Montazeri M, Salsabili N, Pourmand GR (2010). High prevalence of AZFb microdeletion in Iranian patients with idiopathic non-obstructive azoospermia. Indian J Med Res.

[32] Oates RD, Silber S, Brown LG, Page DC (2002). Clinical characterization of 42 oligospermic or azoospermic men with microdeletion of the AZFc region of the Y chromosome, and of 18 children conceived via ICSI. Hum Repord.

